# Association of neural tube defects with maternal alterations and genetic polymorphisms in one-carbon metabolic pathway

**DOI:** 10.1186/s13052-019-0630-1

**Published:** 2019-03-14

**Authors:** Chun-Quan Cai, Yu-Lian Fang, Jian-Bo Shu, Lin-Sheng Zhao, Rui-Ping Zhang, Li-Rong Cao, Yi-Zheng Wang, Xiu-Fang Zhi, Hua-Lei Cui, Ou-Yan Shi, Wei Liu

**Affiliations:** 1 College of Management and Economics, Tianjin University, No.92 Weijin Road, Tianjin, 300072 China; 2 Department of Neurosurgery, Tianjin Children’s Hospital, No.238 Longyan Road, Beichen District, Tianjin, 300134 China; 3 Institute of Pediatrics, Tianjin Children’s Hospital, No.238 Longyan Road, Beichen District, Tianjin, 300134 China; 4 Institute of Pediatrics, Tianjin Children’s Hospital, No.238 Longyan Road, Beichen District, Tianjin, 300134 China; 5 Department of Pathology, Tianjin Children’s Hospital, No.238 Longyan Road, Beichen District, Tianjin, 300134 China; 6 Department of Pediatrics, Tianjin Children’s Hospital, No.238 Longyan Road, Beichen District, Tianjin, 300134 China; 7 Graduate School of Tianjin Medical University, No.22 Qixiangtai Road, Heping District, Tianjin, 300070 China; 8 Graduate School of Tianjin Medical University, No.22 Qixiangtai Road, Heping District, Tianjin, 300070 China; 9 Graduate School of Tianjin Medical University, No.22 Qixiangtai Road, Heping District, Tianjin, 300070 China; 10 Department of Surgery, Tianjin Children’s Hospital, No.238 Longyan Road, Beichen District, Tianjin, 300134 China; 11 School of Basic Medical Sciences, Tianjin Medical University, No.22 Qixiangtai Road, Heping District, Tianjin, 300070 China; 12 Department of Pediatrics, Tianjin Children’s Hospital, No.238 Longyan Road, Beichen District, Tianjin, 300134 China

**Keywords:** Neural tube defects, One-carbon metabolism, Gene, Polymorphism

## Abstract

**Background:**

Neural tube defects (NTDs) are birth defects of the brain, spine, or spinal cord invoked by the insufficient intake of folic acid in the early stages of pregnancy and have a complex etiology involving both genetic and environmental factors. So the study aimed to explore the association between alterations in maternal one-carbon metabolism and NTDs in the offspring.

**Methods:**

We conducted a case-control study to get a deeper insight into this association, as well as into the role of genetic polymorphisms. Plasma concentrations of folate, homocysteine (Hcy), S-adenosylmethionine (SAM), S-adenosylhomocysteine (SAH) and genotypes and alleles distributions of 52 SNPs in 8 genes were compared for 61 women with NTDs-affected offspring and 61 women with healthy ones.

**Results:**

There were significant differences between groups with regard to plasma folate, SAM, SAH and SAM/SAH levels. Logistic regression results revealed a significant association between maternal plasma folate level and risk of NTDs in the offspring. For *MTHFD1* rs2236225 polymorphism, mothers having GA genotype and A allele exhibited an increased risk of NTDs in the offspring (OR = 2.600, 95%CI: 1.227–5.529; OR = 1.847, 95%CI: 1.047–3.259). For *MTHFR* rs1801133 polymorphism, mothers having TT and CT genotypes were more likely to affect NTDs in the offspring (OR = 4.105, 95%CI: 1.271–13.258; OR = 3.333, 95%CI: 1.068–10.400). Moreover, mothers carrying T allele had a higher risk of NTDs in the offspring (OR = 1.798, 95%CI: 1.070–3.021). For *MTRR* rs1801394 polymorphism, the frequency of G allele was significantly higher in cases than in controls (OR = 1.763, 95%CI: 1.023–3.036). Mothers with NTDs-affected children had higher AG genotype in *RFC1* rs1051226 polymorphism than controls, manifesting an increased risk for NTDs (OR = 3.923, 95%CI: 1.361–11.308).

**Conclusion:**

Folic acid deficiency, *MTHFD1* rs2236225, *MTHFR* rs1801133, *MTRR* rs1801349 and *RFC1* rs1051226 polymorphisms may be maternal risk factors of NTDs.

## Background

Neural tube defects (NTDs) are one of the most severe congenital malformations of the central nervous system at birth [1]. NTDs are birth defects of the brain, spine, or spinal cord that occur because of the insufficient intake of folic acid in the early stages of pregnancy. The symptoms of NTDs will vary depending on the specific kind of birth defect. As reported, the average worldwide prevalence is 1 per 1000 living birth [2]. Studies have reported that the prevalence of NTDs exhibit geographical and cultural specifics [3]. The precise etiology is multifactorial mainly involving both environmental and genetic factors. A series of epidemiological studies indicated one possible reason might be the disturbance of one-carbon metabolism pathway [4]. In 1991, Anonymous et al. first clued that periconceptional supplementation of folic acid was able to prevent the recurrence of NTDs [5], which was further confirmed by many other subsequent studies [6]. From these findings, U.S. Public Health Service recommended that all women of childbearing age should be take 400 μg folic acid per day from 4 weeks before impregnation through gestation week 12 in order to prevent first occurrence of a NTDs-affected pregnancy [7].

**Fig. 1:**
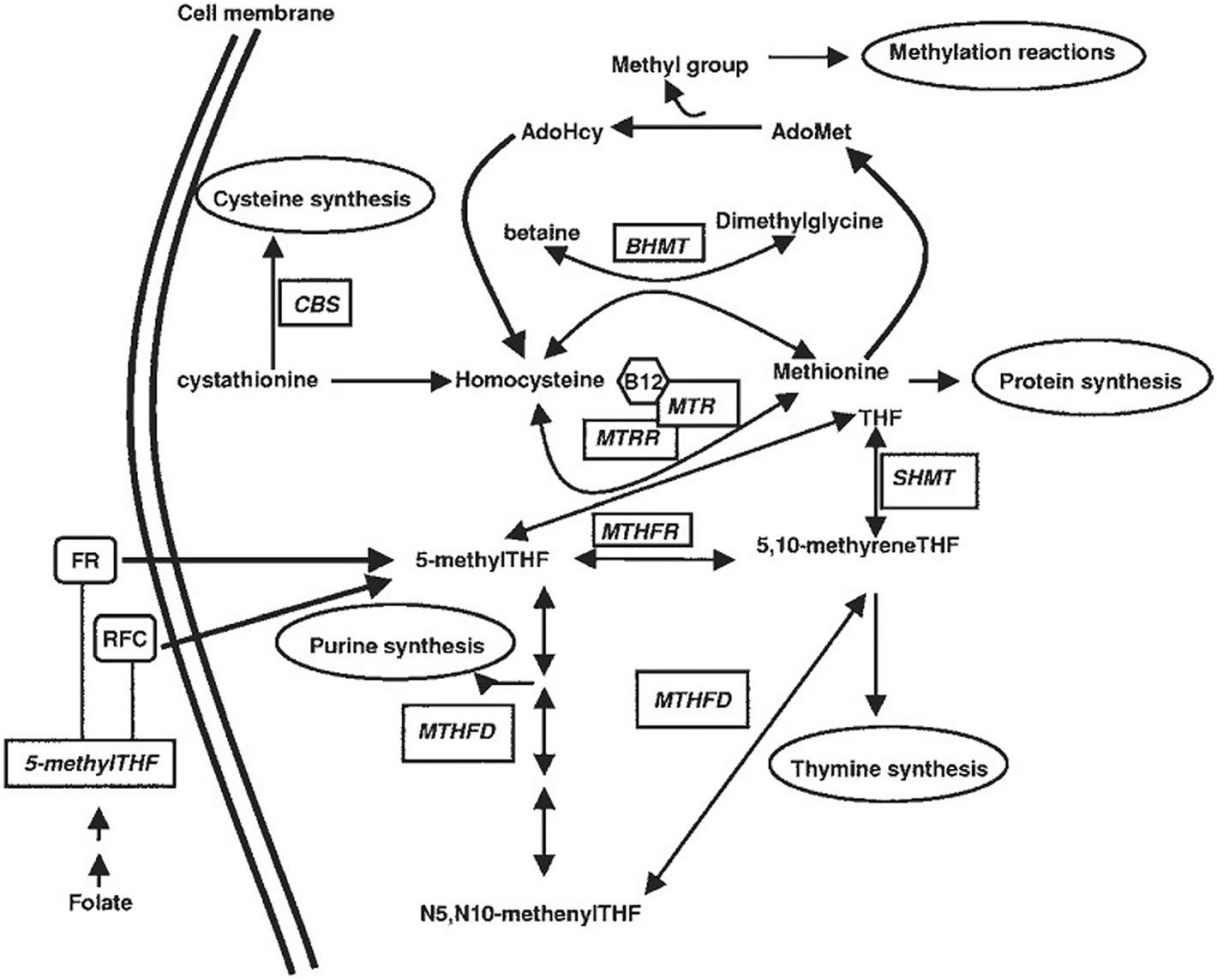
Simplified overview of one-carbon metabolism pathway. *MTHFR*, methylene tetrahydrofolate reductase; *MTHFD1,* methylenetetrahydrofolate dehydrogenase 1; *MTR*, methionine synthase; *MTRR,* methionine synthasereductase; *RFC1,* reduced folate carrier 1; *BHMT,* betaine-homocysteine methyltransferase; CBS, cystathionine beta synthase;*SHMT*, serine hydroxymethyltransferase; AdoHcy, S-adenosylhomocysteine; AdoMet, S-adenosylmethionine

Moreover, a few studies have suggested impaired homocysteine (Hcy) metabolism might be involved in the protective mechanism of folate [8]. Several studies have also revealed that mothers having children with NTDs possess elevated Hcy level [9]. In addition, genetic factors may also invoke NTDs. Many loci in genes involving in the one-carbon metabolism pathway have been found to be associated with the risk of NTDs, such as methylenetetrahydrofolate reductase (*MTHFR*), betaine-homocysteine methyltransferase (*BHMT*), methylenetetrahydrofolate dehydrogenase (*MTHFD1*), methionine synthase (*MTR*), methionine synthasereductase (*MTRR*), and reduced folate carrier (*RFC1*) [10] (Fig. 1)*.*

In this study, we designed a case-control study to investigate the role of maternal alternations and genetic polymorphisms of one-carbon metabolism pathway in the etiology of NTDs. We measured the concentrations of plasma folic acid, Hcy, and the related biological makers. In addition, we genotyped 52 single nucleotide polymorphisms (SNPs) in 8 genes of one-carbon metabolism pathway to investigate the association between genetic polymorphisms and NTDs.

## Methods

### Subjects

The case group consisted of 61 women with NTDs-affected children, which were investigated in the Department of Neurosurgery of Tianjin Children’s Hospital in China from November 2010 to May 2014. The diagnosis of NTDs was based on clinical manifestations and images. The control group was composed of 61 age-matched women who had the same ethnic background, but no children affected by NTDs or any other congenital malformations. All participants were from Chinese Han population in the North, Northwest, and Northeast of China. The work was approved by the Tianjin Children’s Hospital Ethics Committee and informed consent was obtained from all subjects.

### Sample preparation and biomarker measurement

Peripheral blood samples were collected from all participants in the study after obtaining their consent. The tubes used to collect peripheral blood contained EDTA as anticoagulation. Plasma was separated and stored at − 20 °C. DNA was extracted from blood samples using the DNA Extraction Kit (Tiangen, China) according to the manufacturer instruction. The collected DNA samples were stored at − 80 °C before use.

Firstly, folic acid concentration was measured using an Access® Immunoassay system (Beckman Coulter, Krefeld, USA). Then, the Hcy concentration was measured using a fluorescence polarization enzyme-based immunoassay (FPIA, Abbott Laboratories, USA). Finally, SAM and SAH were assayed by the multiple reaction monitoring.

### Selection of candidate SNPs

Eight genes were selected in folate and Hcy metabolism pathway according to relevant references, including *BHMT*, *MTHFD1*, *MTHFR*, *MTR*, *MTRR*, *RFC1, SHMT1,* and thymidylate synthase (*TYMS*). SNPs were searched and selected using the Haploview4.2 software in HapMap database according to the following criteria: Han population of the Northern China; HWpval (*P* value of Hardy-Weinberg equilibrium test) > 0.05; MAF (Minor Allele Frequency) ≥0.05. Subsequently, a further analysis was employed to select proper SNPs using functional prediction Website and dbSNP database, and 52 SNPs from 8 candidate genes were selected.

### Genotyping

Fifty-two SNPs of 8 genes were genotyped via the Sequenom-based Mass ARRAY assay. Then, the data of genotype and allele distributions were incorporated and analyzed via the Filemaker Pro Database, which is a cross-platform relational database application from FileMaker Inc. (https://www.filemaker.com).

### Statistical analysis

All statistical analyses were realized via the SPSS 17.0 software. Measurement data were presented as mean ± standard deviation values, and comparison among groups was conducted by the *t*- or rank-sum tests. Frequency and rate values were used to denote count data, while comparison among groups was provided by the Chi-square or Fisher’s exact tests. The Hardy-Weinberg equilibrium test was conducted in the control group by the Chi-square tests in order to detect group representation. Statistical significance was accepted at *P* ≤ 0.05. Odds ratio (OR), with 95%-confidence interval*,* was calculated to estimate the relative risk of different genotype combinations. Online SIFI software (http://sift.jcvi.org) and PolyPhen-2 software (http://genetics.bwh.harvard.edu/pph2) were used to predict the potential functions of these pathogenic mutations.

## Results

### Maternal biomarkers and risk of NTDs in the offspring

The biomarkers used for cases and controls are listed in Table 1. As can be observed, plasma folate, SAM concentrations and SAM/SAH ratios were significantly lower in cases than that in controls, and the differences showed statistical significance (*P* < 0.001). The SAH concentration of the cases was significantly higher than that of the controls, and the difference showed a statistical significance (*P* < 0.001). Logistic regression results revealed a significant association between maternal folate level and the risk of NTDs in the offspring (Table 2).

**Table 1:** Distribution of biological makers of folate metabolism in cases and controls

	Case(*n* = 61)	Control(*n* = 61)	t values	*P* values
Plasma folate(nM)	7.7 ± 1.7	9.4 ± 1.6	−5.687	< 0.001*
Plasma Hcy(μM)	12.3 ± 3.2	11.3 ± 2.3	1.982	0.050
Plasma SAM(nM)	49.9 ± 3.0	52.8 ± 3.2	−5.164	< 0.001*
Plasma SAH(nM)	12.9 ± 1.7	10.1 ± 1.9	8.578	< 0.001*
SAM/SAH	3.9 ± 0.6	5.4 ± 1.0	−10.046	< 0.001*

Note:*P value< 0.05 indicates that these biological makers showed a significant difference between case and control groups

**Table 2:** The results of logistic regression analyses between case and control groups

	β	*P* values	Exp(β)	95%Confidence interval for β	
				Lower bound	Upper bound
Plasma folate	0.837	0.000*	2.310	1.453	3.674
Plasma Hcy	−0.244	0.117	.783	0.577	1.063
Plasma SAM	0.183	0.543	1.201	0.666	2.165
Plasma SAH	0.185	0.879	1.203	0.112	12.963
SAM/SAH	3.368	0.295	29.020	0.053	15,927.004
constant	−31.100	0.046	.000		

Note:*P value< 0.05 indicates that plasma folate level showed a significant difference between case and control groups

β: logistic regression coefficient; Exp(β) e^β^: odds ratio value

### Hardy-Weinberg equilibrium

The genotype frequencies of the vast majority of SNPs accorded with Hardy-Weinberg equilibrium in the control group (*P >* 0.05), except for the following four loci: *BHMT* rs7700970, *MTHFR* rs2066470, *MTRR* rs1532268, and *MTRR* rs1802059.

### Genotype frequencies of various SNPs in cases and controls

Because 4 SNPs did not conform to Hardy-Weinberg equilibrium, they were excluded from the statistical analysis, so that the total number of SNPs analyzed in our study was reduced to 48. The results showed that 4 SNPs of mothers were associated with the susceptibility of NTDs in the offspring (*P* < 0.05), including *MTHFD1* rs2236225, *MTHFR* rs1801133, *MTRR* rs1801394, *RFC1* rs1051266. The observed frequencies of various genotypes and alleles of *MTHFD1* rs2236225 polymorphism are listed in Table 3. The case group had a higher frequency of 1958 GA genotype than the control one, and the difference showed a statistical significance (OR = 2.600, 95% CI: 1.227–5.529), and so did the frequency of A allele (OR = 1.847, 95% CI: 1.047–3.259).

**Table 3:** Genotype and allele frequencies of the *MTHFD1* G1958A polymorphism in case and control groups

Genotype	Case(N = 61)	Control(*N* = 61)	*χ*^2^ values	OR	95%CI	*P* values
GG	23(37.7)	37(60.7)		1		
GA	34(55.7)	21(34.4)	6.331	2.600	1.227~5.529	0.012*
AA	4(6.6)	3(4.9)		2.145	0.440~10.464	0.427^**^
G	80(65.6)	95(77.9)		1		
A	42(34.4)	27(22.1)	4.547	1.847	1.047~3.259	0.033*

Note: *P value< 0.05 indicates that the genotype and allele distributions of *MTHFD1* G1958A polymorphism exhibit a significant difference between case and control groups

^**^P value are calculated using Fisher’s exact test

In terms of the *MTHFR* rs1801133 polymorphism, the risk of offspring affected by NTDs from mutated homozygous mothers (677 TT genotype) was significantly higher than that from mothers with 677 CC genotype (OR = 4.015, 95% CI: 1.271–13.258). In case of heterozygous mothers, an increased risk of offspring-NTDs was observed (OR = 3.333, 95% CI: 1.068–10.400). A pronounced association was found between the maternal 677 T allele and NTDs in the offspring (OR = 1.798, 95% CI: 1.070–3.021), as is shown in Table 4.

**Table 4:** Genotype and allele frequencies of the *MTHFR* C677T polymorphism in case and control groups

Genotype	Case(N = 61)	Control(*N* = 61)	*χ*^2^values	OR	95%CI	*P* values
CC	5(8.2)	15(24.6)		1		
CT	30(49.2)	27(44.3)	4.559	3.333	1.068~10.400	0.033*
TT	26(42.6)	19(31.1)	5.963	4.105	1.271~13.258	0.015*
C	40(32.8)	57(46.7)		1		
T	82(67.2)	65(53.3)	4.945	1.798	1.070~3.021	0.026*

Note: *P value< 0.05 indicates that the genotype and allele distributions of *MTHFR* C677T polymorphism exhibit a significant difference between case and control groups

Table 5 presents the data on *MTRR* rs1801394 polymorphism. There was a statistical difference between case and control groups with respect to 66 G allele (OR = 1.763, 95% CI: 1.023–3.036).

**Table 5:** Genotype and allele frequencies of the *MTRR* A66G polymorphism in case and control groups

Genotype	Case(N = 61)	Control(N = 61)	*χ*^2^values	OR	95%CI	*P* values
AA	22(36.1)	33(54.1)		1		
AG	31(50.8)	24(39.3)	2.947	1.938	0.908~4.136	0.086
GG	8(13.1)	4(6.6)	2.833	3.000	0.805~11.184	0.092
A	75(61.5)	90(73.8)		1		
G	47(38.5)	32(26.2)	4.212	1.763	1.023~3.036	0.040*

Note: *P value< 0.05 indicates that the genotype and allele distributions of *MTRR* A66G polymorphism exhibit a significant difference between case and control groups

For the *RFC1* rs1051266 polymorphism, genotype and allele frequencies are presented in Table 6. The case group had a higher frequency of mutated AG genotype than the control one, and the difference showed a statistical significance (OR = 3.923, 95% CI: 1.361–11.308).

**Table 6:** Genotype and allele frequencies of the *RFC1* A80G polymorphism in case and control groups

Genotype	Case(N = 61)	Control(N = 61)	*χ*^2^values	OR	95%CI	*P* values
AA	6(9.8)	17(27.9)		1		
AG	36(59.0)	26(42.6)	6.863	3.923	1.361~11.308	0.009*
GG	19(31.1)	18(29.5)	3.725	2.991	0.964~9.276	0.054
A	48(39.3)	60(49.2)		1		
G	74(60.7)	62(50.8)	2.392	1.492	0.898~2.479	0.122

Note: *P value< 0.05 indicates that the genotype and allele distributions of *RFC1* A80G polymorphism showed significant differences between case and control groups

### Bioinformatics analysis

Mutations were analyzed by PolyPhen-2 online software, which predicted the pathogenic nature of missense mutations. The prediction could define the putative role of missense variants and could assess whether they were probably/possibly damaging or benign [11]. Our results manifested that *MTHFR* rs1801133 and *MTRR* rs1801394 mutations were “probably damaging”, suggesting their potential involvement with NTDs.

SIFT software was used to analyzed the possibility on the pathogenic properties of mutations by comparing the mutant and normal variants. Sift scores of point mutation were calculated for weighting the mutations as damaging or tolerated [12]. Our results suggested *MTHFR* rs1801133 and *MTRR* rs1801394 mutations as “damaging” variants.

## Discussion

NTDs are recognized to have a complex etiology, mainly involving both genetic and environmental factors. Several observations supported the opinion that genetic factors mainly involve the planar cell polarity (PCP) signaling pathway [13, 14], folate metabolism pathway [15], and glycometabolism pathway [16]. Nuritional factors is the most common environmental factor influencing susceptibility to disease. Folic acid consumption may help to decrease the effects of the low enzymatic activity of one-carbon metabolism pathway [17]. Related studies have verfied that the process of absorption and biotransformation of folic acid to its active form was saturated at doses in region of 200~400 μg of folic acid. The indicator, erythrocyte folate concentration, has been used to assess longer-term folic acid intake and storage [18]. Recent investigations have demonstrated that erythrocyte folate concentration above 1000 nmol/l was the level required for optimal NTDs prevention. Subsequent studies have further comfirmed that a period of more than 12 weeks with 400 μg per day was needed to achieve the reported level of erythrocyte folate concentration [19]. A number of epidemiological studies have further confirmed that folic acid fortification could prevent NTDs to a large extent [18, 20].

The increased SAH was also reported as an effective inhibitor of the cellular methyltransferase activity [21, 22]. Low methionine and SAM concentrations in combination with increased SAH and adenosine concentrations have been reported to be associated with reduced methylation capacity [23]. Our results strongly indicated that mothers with NTDs-affected offspring had higher SAH concentrations, and lower plasma folate and SAM concentrations, as compared to mothers with no such children. Then, logistic regression results revealed a significant association between maternal folate level and the risk of NTDs in the offspring. This implied that decreased folic acid was a maternal risk factors for NTDs in the offspring.

The enzyme activity of *MTHFD1* was reported to play a crucial role in one-carbon metabolism by providing folate cofactors for DNA synthesis and cellular methylation reactions [24]. Furthermore, *MTHFD1* rs2236225 (G1958A) has been studied in several populations as a functional exonic SNP. A number of studies indicated that *MTHFD1* G1958A polymorphism has a significant association with risk of NTDs in both NTDs patient and maternal groups [25]. Our results were consistent with this conclusion, showing a significant association between mothers with AG genotype and A allele, and NTDs in the offspring.

The association between maternal *MTHFR* gene rs1801133 (C677T) polymorphism and NTDs susceptibility was controversial in different populations worldwide [26, 27]. Our research on NTDs mothers showed a significant association with NTDs in the offspring, suggesting that *MTHFR* C677T polymorphism was a maternal risk factor with a higher probability for mothers to have NTDs offspring. These data were consistent with those of other researchers who reported a higher prevalence in case mothers as compared to controls [28]. PolyPhen-2 and SIFT softwares prediction supported the result, suggesting the *MTHFR* C677T as a possibly damaging variant.

Several studies from various populations have indicated that *MTRR* rs1801394 (A66G) polymorphism was a likely maternal risk factor for development of NTDs pregnancy [29]. Our study revealed a higher risk of NTDs among mothers with G allele, as compared to those with A allele. This implied that *MTRR* rs1801394 polymorphism was a maternal risk factor for NTDs, which complies with the earlier obtained results. PolyPhen-2 and SIFT softwares prediction supported the result, suggesting the *MTRR* A66G as a possibly damaging variant.

*RFC-1* is a vital transporter for the folate substrate 5-methyltetrahydrofolate. This transporter is of particular importance during embryonic development in transporting folate across the placenta [30]. *RFC-1* rs1051266 polymorphism (A80G) might impair folate transport from maternal blood to the fetus, which could be a maternal risk factor of NTDs [31, 32]. Our findings confirmed a statistically significant difference between *RFC-1 80AG* genotype in cases and controls. The maternal *RFC-1* rs1051266 polymorphism was considered as a risk factor for NTDs in the offspring.

## Conclusions

Based on the results obtained, a conclusion can be drawn that the maternal folate supplementation can reduce the incidence of NTDs in the offspring, while *MTHFD1* rs2236225, *MTHFR* rs1801133, *MTRR* rs1801394and *RFC-1* rs1051266 polymorphisms may increase the risk of NTDs in Han Chinese population of Northern China. Due to the limited sample size in our study, further investigations with a larger sample size and more functional analysis have to be conducted, in order to get a deeper insight into the etiology of NTDs.

## Abbreviations

AdoHcy: S-adenosylhomocysteine
AdoMet: S-adenosylmethionine
BHMT: Betaine-homocysteine methyltransferase
CBS: Cystathionine beta synthase
Hcy: Homocysteine
MTHFD1: Methylenetetrahydrofolate dehydrogenase
MTHFR: Methylenetetrahydrofolate reductase
MTR: Methionine synthase
MTRR: Methionine synthasereductase
NTDs: Neural tube defects
RFC1: Reduced folate carrier
SAH: S-adenosylhomocysteine
SAM: S-adenosylmethionine
SHMT: Serine hydroxymethyltransferase
SNPs: Single nucleotide polymorphisms
TYMS: Thymidylate synthase
